# Leprosy elimination - Still a long way to go

**DOI:** 10.1590/1516-3180.2018.0345021019

**Published:** 2020-02-18

**Authors:** João Avancini, Maria Ângela Bianconcini Trindade, José Antonio Sanches

**Affiliations:** I MD. Supervisor, Dermatology Division, Hospital das Clinicas HCFMUSP, Faculdade de Medicina, Universidade de Sao Paulo, Sao Paulo, (SP), Brazil.; II MD, PhD. Researcher, Institute of Health, Hospital das Clinicas HCFMUSP, Faculdade de Medicina, Universidade de Sao Paulo, São Paulo (SP), Brazil.; III MD, PhD. Full Professor, Dermatology Department, Hospital das Clinicas HCFMUSP, Faculdade de Medicina, Universidade de Sao Paulo, São Paulo (SP), Brazil.

Elimination of leprosy as a public health problem at the global level was considered achieved in the year 2000, when the registered prevalence reached less than one case of the disease per 10,000 inhabitants.^1^ The Global Leprosy Strategy 2016-2020 published by the World Health Organization (WHO) established a goal of further reduction of the leprosy burden. Moreover, the primary target of the strategy moved from elimination towards an emphasis on early detection, reduction of grade-2 disabilities (i.e. visible impairments/deformities at the time of diagnosis) and reduction of transmission.^1^

Although detection of new cases has shown a modest decline over the last five years, the grade-2 disabilities rate among new cases has remained almost static, thus indicating a continued delay in detection.^1^^,^^2^^,^^3^ Efforts have been made in Brazil regarding early detection, such as active case search and continuous medical education for primary care workers, using tools such as e-learning and telemedicine.^4^

**Figure 1.**
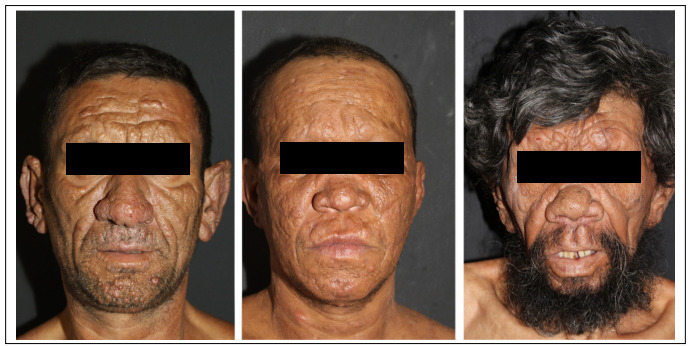
Advanced multibacillary leprosy presenting with madarosis and diffuse face infiltration.

**Figure 2.**
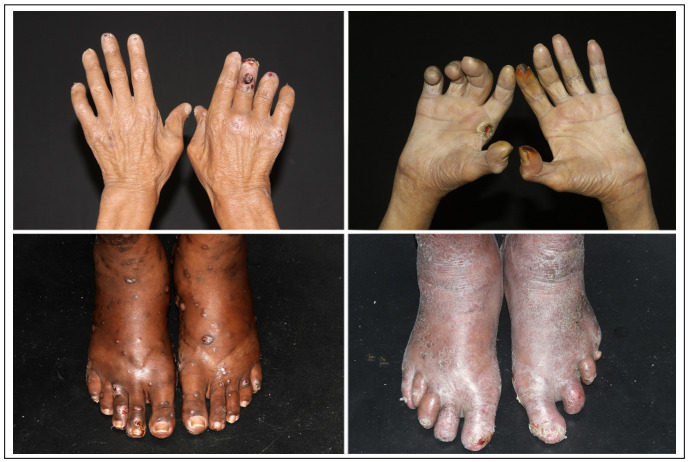
Lesions on extremities leading to reabsorption of distal phalanges and skin ulcers relating to the lack of protective sensitivity; numerous lepromas in lower left image.

The state of Sao Paulo had a prevalence rate of 0.36 cases of leprosy/10,000 inhabitants in 2017. Therefore, leprosy is not considered endemic in this state.^5^ Nonetheless, we continue to make diagnoses of multibacillary patients showing grade-2 disabilities in our hospital, which is a quaternary-level care facility in the largest city in Brazil. We present images of multibacillary individuals diagnosed in our hospital over the last five years (Figures 1 and 2). All of these patients authorized the use of their images. As can be seen, these patients presented numerous cutaneous and neurological features that made it mandatory to consider leprosy at least as a differential diagnosis in any medical consultation. Furthermore, most of these patients received the diagnosis only when they reached our service.

Out of 121 patients diagnosed with leprosy in our hospital, 20 (17.2%) already presented grade-2 disabilities at the time of the diagnosis, while 56 (46.3%) had grade-1 disabilities. Most of our patients were not born in the city of São Paulo, but they had been living in the city for at least ten years. In the state of São Paulo, within the last five years, a total of 515 patients, corresponding to 13% of the new diagnosed cases, presented grade-2 disabilities at the time of the diagnosis. Since multibacillary patients are responsible for sustaining the endemic status of leprosy in Brazil, we can conclude that we are still failing to reach the goal of early diagnosis.

The possible causes of late diagnosis are the following: (i) lack of education: most of these patients had never completed their formal schooling; (ii) poor sanitary conditions: some of these patients were homeless or lived in slums; (iii) history of alcohol abuse; (iv) difficulties in accessing healthcare; and (v) lack of suspicion of the diagnosis of leprosy among doctors: even when such patients reach a healthcare service, it is rare for leprosy to be suspected.

Despite all the efforts by healthcare providers and despite healthcare policies that focus on the disease itself, political measures towards providing social advances remain necessary. If the abovementioned causes of late diagnosis persist, undiagnosed multibacillary patients will still face long delays before proper treatment is implemented and will probably end up transmitting the disease, thus sustaining its endemic status. Evaluation of households is still probably the most accessible way to conduct an active search for cases, especially in endemic areas.
